# Equity in international health research collaborations in Africa: Perceptions and expectations of African researchers

**DOI:** 10.1371/journal.pone.0186237

**Published:** 2017-10-16

**Authors:** Nchangwi Syntia Munung, Bongani M. Mayosi, Jantina de Vries

**Affiliations:** 1 Department of Medicine, University of Cape Town, Cape Town, South Africa; 2 Groote Schuur Hospital, Cape Town, South Africa; Iowa State University, UNITED STATES

## Abstract

**Introduction and method:**

Africa is currently host to a number of international genomics research and biobanking consortia, each with a mandate to advance genomics research and biobanking in Africa. Whilst most of these consortia promise to transform the way international health research is done in Africa, few have articulated exactly how they propose to go about this. In this paper, we report on a qualitative interviewing study in which we involved 17 genomics researchers in Africa. We describe their perceptions and expectations of international genomics research and biobanking initiatives in Africa.

**Results:**

All interviewees were of the view that externally funded genomics research and biobanking initiatives in Africa, have played a critical role in building capacity for genomics research and biobanking in Africa and in providing an opportunity for researchers in Africa to collaborate and network with other researchers. Whilst the opportunity to collaborate was seen as a benefit, some interviewees stressed the importance of recognizing that these collaborations carry mutual benefits for all partners, including their collaborators in HICs. They also voiced two major concerns of being part of these collaborative initiatives: the possibility of exploitation of African researchers and the non-sustainability of research capacity building efforts. As a way of minimising exploitation, researchers in Africa recommended that genuine efforts be made to create transparent and equitable international health research partnerships. They suggested that this could be achieved through,: having rules of engagement, enabling African researchers to contribute to the design and conduct of international health projects in Africa, and mutual and respectful exchange of experience and capacity between research collaborators. These were identified as hallmarks to equitable international health research collaborations in Africa.

**Conclusion:**

Genomics research and biobanking initiatives in Africa such as H3Africa have gone some way in defining aspects of fair and equitable research collaborations in Africa. However, they will need to strive at achieving equitable health research collaborations if they truly aim at setting a gold standard for how international health research should be conducted in Africa.

## Introduction

In 1990, the Commission for Health Research and Development (COHRED) reported that less than 10% of global health research funding was spent on health conditions that account for 90% of the global disease burden [1]. This led to an outcry that if it remained the status quo, global inequities in health will persist [2]. COHRED also highlighted the important role health research could play in reducing global health inequities. This has since led to an increase in the number international health research projects in Africa [3, 4]. International health research is research conducted in low and middle income countries (LMICs) with funding from institutions and organisations in high income countries (HICs). Clinical trials have dominated the international health research landscape, however there is currently a growing number of genomics research and biobanking projects in Africa which are funded by institutions and organizations in HICs [5–7]. Examples include: the African Genome Variation Project; Human Heredity and Health in Africa (H3Africa) Consortium; and the Bridging Biobanking and Biomedical Research across Europe and Africa (B3Africa) initiative. The explanation for the growing interest in genomics research and biobanking in Africa is that it will facilitate cutting-edge health research on African populations; prevent a genomics divide between HICs and Africa; reduce global health inequities [8, 9] or–more skeptically–generate evidence that will be of benefit to genetically homogeneous populations [10, 11].

Despite the potential of international health research to reduce global inequities in health and research, it also has the potential to exploit research participants and African researchers [12, 13]. In the late 1990s, for example, there was a spark of debates on the ethics of international health research in Africa [14–17], a number of which highlighted the impact social and economic differences between HICs and LMICs could impact on international health research. The debates also suggested that the asymmetrical nature of these partnerships tend favor research collaborators in HICs [14–19] while their African counterparts ended up as sample collectors [20–22].

Contemporary genomics research and biobanking initiatives in Africa are promising to transform the way international health research has typically been structured in Africa. They hope to minimize the possibility of exploitation of African researchers [9, 23–25]. Their plan is to go beyond the traditional practice of collecting samples in Africa and conducting the scientific analyses outside of the continent, to one that fosters equitable research collaborations. While in theory, the intention to foster equitable research partnerships is laudable, these promises remain largely unchecked. There is a need to go beyond the documented promises and explore whether and how genomics research and biobanking initiatives in Africa are going about realizing these goals, and how successful their efforts are perceived to be by their LMIC partners. In this paper, we document the perceptions and expectations of genomics researchers in Africa, on the benefits of externally funded genomics research and biobanking initiatives in Africa. This study identifies challenges pertaining to equity in research collaborations which HIC partners should consider when they engage in international health research in Africa.

## Methods

We adopted a qualitative research methodology. Between September 2014 and June 2015, 17 face-to-face semi-structured interviews were conducted with genomics researchers in Africa. Purposive sampling [26] was used to select research participants. Research participants were genomics researchers based in an African research institution and who were directly involved in an international genomics research and biobanking project. Principal investigators and co-principal investigators within the H3Africa Consortium were first invited to be part of the study. We selected this group of participants because, we are part of the consortium and therefore were certain that participants were actively involved in international health research projects. After interviews with these group of researchers, we invited non-H3Africa researchers, involved in other collaborative genomics research or biobanking initiatives in Africa, to be part of the study. The reason was to attain data saturation [27]. H3Africa is an African-led genomics research and biobanking initiative with funding from the National Institute of Health, USA and The Wellcome Trust, United Kingdom [9].

Research ethics clearance was obtained from the Faculty of Health Sciences Health Research Ethics Committee, University of Cape Town (HREC-Ref-618/2014). Written informed consent was obtained from all research participants before the start of the study.

Of the 17 interviewees, 14 were part of the H3Africa consortium while 3 were not involved in H3Africa. Interviewees were based in 8 African countries and included biomedical and social scientists. At the time of the interviews, 13 of the 17 researchers were principal investigators or co-principal investigators for a genomics research and/or biobanking project in Africa, while four were research scientists.

The researchers were asked questions about their experiences of being part of an international health research consortium; what they perceived to be the benefits and risks; and their recommendations on how international health research should be done in Africa. All interviews were conducted in English and lasted approximately one hour.

Audio recorded interviews were transcribed verbatim. Iterative data analysis started immediately after the first few interviews and continued throughout the data collection phase. This allowed for the identification of themes and patterns that were emerging from the data, which were further probed in subsequent interviews [28]. Data collection continued until we reached saturation point where no new patterns or categories were emerging from the interviews [27, 29]. To facilitate analysis and management, we usedNVivo 10 [30], a text-based data analysis software. We performed inductive thematic analysis [31] of the interview transcripts with the objective of identifying, examining and recording patterns of meaning across the dataset [32, 33]. These patterns were identified through a process of data familiarization, data coding and the development and revision of themes and models [34, 35]. NSM did the initial coding for the first few interview transcripts. Following the first round of coding, codes were discussed with JDV. NSM and JDV then separately coded several transcripts to establish validity of the coding scheme and its application. Differences in coding were discussed and the coding scheme was adapted to clarify ambiguities. NSM continued to code the entire dataset and interpreted the coded data. Challenges and evolving data interpretations were discussed regularly with JDV.

## Results

In the interviews, as genomic researchers shared their perceptions, expectations and recommendations for international health research partnerships in Africa, a number of significant insights emerged.

All interviewees were of the view that externally funded genomics research and biobanking initiatives in Africa provided a platform for researchers in Africa to to collaborate, on a large scale, with researchers from different disciplinary backgrounds and from different parts of the world. This, they hoped, will play a critical role in narrowing the existing gap in genomics research capacity between HICs and LMICs and in preventing a possible genomic divide between Africa and HICs.

This opportunity will allow us to bring genomics in Africa to a level that is comparable to other parts of the world and it could potentially lead to new therapies and treatment strategies that are relevant, needed in Africa for which we couldn’t do by using results from genomics research from other parts of the world either because of the differences in the inheritability of the different conditions in Africa compared to other parts of the world or because of the gene-environment interactions in different parts of Africa and that is different from what you see in other parts of the world. (R-01)

A view that has also been articulated by other researchers in Africa [36, 37]. In one case, An African researcher said that without international health research, researchers in Africa, would be sitting all day in their offices, ‘reading newspapers’ instead of doing research [38]. Although all our interviewees cited the opportunity to collaborate as a benefit, some concurrently stressed that these collaborations carry mutual benefits for all partners, including collaborators in HICs.

This part of the world has not got the necessary equipment to carry out certain experiments. The way to go is to collaborate. By collaborating, each individual group brings in particular expertise. So if we are working on African genomics then the first thing that the African scientists can bring in is the African genome and then the other people can come in from other sides and bring in their expertise and all that. (R-17)

Recognition of this mutual benefit is important for international health research partnerships in Africa. International health research has often been portrayed as a form of development aid or altruistic philanthropy, where the receiver is expected to show some gratitude to the giver [37, 39]. This has led to situations whereby HIC collaborators had said to their African collaborators that they were only hired to do the research and not to be involved in key decision making activities [38]. This is problematic in that it highlights international health research as a patronizing and neocolonial activity. Nevertheless, African researchers are increasingly judging these collaborations as being of mutual benefit to all partners, and as a result want their HIC collaborators not to treat them as employees but as partners. For example, some African researchers are now willing to ask their collaborators, upfront, without fear of losing a funding opportunity, “*Do you want me to work with you or to work for you?*” [38]. These are indeed good signs for international health research in Africa because when research collaborations are seen as mutually beneficial to all partners, they are arguably closer to being equitable. It is therefore imperative that HIC partners involved in these collaborations recognize the implications this may have in achieving equitable collaborations when they setup international health research partnerships in Africa.

### Fears of exploitation of African researchers

The recognition by African researchers that international health research partnerships are mutually beneficial has however not allayed fears of exploitation of African researchers by their partners in HICs. Despite being generally supportive of African genomics research and biobanking initiatives in Africa, all our interviewees expressed concerns that they may end up being exploited in these collaborations. These fears were primarily shaped by past experiences of exploitation of African scientists.

It could be that African researchers, as it has happened before, are just being used for collecting materials and that is a very big potential [for exploitation] because if you don’t have capacity to analyse, make sense out of it, you just collect the material and the data and send it to people who can analyse and that could be a benefit not only for publication of an article but longer term benefit of patents and discoveries… Those are some of the potential risks. (R-07)

The fear of exploitation is made worse by uncertainty about sustainability of genomics research and biobanking projects in Africa. One skeptical view explaining the growing interest in African samples is that access to Africa’s rich human genetic diversity [9] makes the continent a choice destination for population genomics studies [10]–though not always to the benefit of African populations, patients or researchers. Interviewees expressed concerns that they were unsure of what will happen to their research projects once the current funding period is over and they no longer have funds to enable them use the samples and data collected in the initial stages of these genomics research and biobanking projects.

For example, about two thirds of our interviewees mentioned that whilst their collaborators in HICs have the capacity and resources to continue research on stored samples and the data that were jointly obtained during the funded collaboration, they would not have access to similar research funds. The risk, therefore, is that though initial studies may seek to establish more equitable forms of collaboration, African researchers may still be marginalized in subsequent research projects.

Our capacity just to handle the data first, to analyse them, to handle the samples, to analyse them or even to have research means in terms of funding, to take advantage of those samples or data is by far limited as compared to our international partners that are in the same project. So what will happen if we design our own research questions to take advantage of the biobank? Will we still be able to have the amount of funding as we are having now in [consortium name] to respond to our own question? Otherwise you will see that after [consortium name] the biobanks will benefit more the international scientists because they will have more resources and we might end up working in collaboration again with international scientists on their agenda and not our agenda (R-05)

This researcher highlights not only the importance of equity to access to samples, data and funding to support their continued work but also that researchers in Africa want to be able to drive their own research agendas. A theme is further discussed below. The inequitable nature of international health research collaborations have led to calls for funders of international health research to build capacity for health research in Africa as well as for HICs partners to engage with their African collaborators in ways that build mutual trust and respect [37, 39–43]. In the interviews, we asked the researchers how these fears of exploitation may be assuaged. Their responses can be grouped into two broad categories: Equitable research partnerships and research capacity building.

### Establishing equitable research partnerships

The nature of research collaborations between LMIC researchers and HIC researchers is often uneven in terms of access to research funding, research resources and involvement in decision making [13, 37, 44]. These power imbalances have made African research partners to oftentimes remain silent about worrying inequalities [38]. As we described above, a two-thirds majority of our interviewees expressed the desire to be in equitable research partnerships. They also had suggestions for how equitable collaborations may be achieved. This included: setting the “rules” of engagement, involvement of African researchers in decision making and African leadership of international health research projects.

#### Setting the “rules” of engagement

The first suggestion advanced by our interviewees as one of the major ways of achieving transparent and equitable research partnerships is having “rules of engagement” between collaborators.

I think importantly around developing the rules of engagement, so, making sure that Africa benefits from the research, not just [consortium name] research but that this kind of work done in Africa, I mean, setting the pace, that this is how genomics research should be done in Africa so that Africa benefits. (R-12)

Slightly over half of our interviewees who expressed the desire for equitable research partnerships specified that the rules of engagement need to be defined before the start of the collaborations. For these interviewees, it is critical that at an early stage of the collaboration, all partners have an idea of what is expected of each collaborator (both LMIC and HIC partner). Recently, some authors have recorded how collaborators in HICs have dictated what needs to be done in research collaborations and in some cases have said their African collaborators were hired to do research and should implement as requested [38]. The resistance of our interviewees to sign up to this way of collaborating may therefore be evidence that they are fighting what Okeke has termed the ‘little brother effect’ in African biosciences [42], that is, north-south partnerships that are a mirror of paternalism and colonial hegemony even though the HIC partners may have initially displayed good intentions at the beginning.

#### Involvement of African researchers in decision making

A second way in which more than half of the interviewees felt equitable partnerships may be achieved was for researchers in Africa to be actively involved in decision making processes in the consortia. As one of the interviewees stated, these collaborations can only be considered fair if they are treated as equal by their collaborators through being involved in decision making activities.

There should also be regulatory procedures that will make sure that the African scientists are involved and are central to any decision involving the use of the samples and are actually involved in the publications and the intellectual property that emanates from such processes. African scientists should be at the centre of all of this. So it is not disadvantaging anyone (R-06)

Again, these are indications that African researchers do not want to be considered as hired workers but as equal research partners. From the interviews, it is clear that African researchers want to be involved in making decisions on the use of samples collected as part of genomics research and biobanking in Africa. Genomics research and biobanking are generally characterized by the sharing of samples and data and most times the cross-border movement of samples for the purposes of analysis.

#### African leadership of genomics research and biobanking in Africa

The perceived fears of exploitation has also led to an expressed yearning for an African leadership of international health research consortia in Africa. More than half of the interviewees recommended that African researchers should be at the forefront in the design and conduct of genomics research and biobanking projects in Africa. As one interviewee said, the story of Africans needs to be told by Africans. Interviewees who articulated a preference for African leadership of research projects in Africa, were clear that they wanted to be involved in all phases of the studies including research design and analysis.

First of all, African scientists should be involved in doing this research. It should not be research that comes from outside Africa, being implemented by non-Africans researchers in Africa. (R-14)

Well I think that it [African ownership] is a good idea in the sense that we will not have a stranger telling us our story that is the positive part of it. (R-16)

This perception is similar to what Okwaro and Geissler reported in a study on scientific collaborations in HIV research whereby researchers in an African research institution expressed interest in leading projects in Africa and criticized the use of the term “local PI” as a way of demonstrating local leadership of projects whilst in actual facts ‘local PIs’ are often only used to implement projects designed by northern collaborators [38]. Our interviewees objected to such tokenistic involvement in decision-making and were rather expressing a desire for substantive involvement.

The desire for equitable research partnerships does not necessarily mean that African researchers thought all collaborators needed to be equal. In fact all the researchers acknowledged that there were disparities in resources and capacity for genomics research between HIC and LMIC investigators, but indicated that this should not stop them from active and equal participation in network activities as their collaborators in HICs.

We must have a critical mass of people who can talk the language of genomics, who can, you know, when there is collaboration their participation is equal. Maybe not in terms of funding but certainly in terms of intellectual contribution. (R-03)

The acknowledgment of these inequalities and how it may impact on the success of international health research in Africa is a step towards achieving equitable international health research partnerships. It is also contrary to what may have been expected a few years back [38, 42], and shows the willingness and eagerness of African researchers to take up roles that go beyond sample collection.

### Research capacity building

Besides building equitable research partnerships as a way of minimizing the possibility of exploitation of African researchers, all our interviewees also said research capacity building will play a big role in minimising exploitation of African researchers and in achieving equitable research partnerships. Some ongoing genomics research and biobanking initiatives in Africa are promising to build research capacity in Africa to ensure that genomics research is done in Africa by Africans [9, 45, 46]. But whilst building health research capacity in LMICs is key to promoting justice in global health research [47], the central question is which type of capacity building efforts can foster equitable genomics research and biobanking collaborations in Africa. When tour interviewees talked about capacity building for genomics research and biobanking in Africa, it was in three major areas: building of human capacity, infrastructural capacity building and the sustainable access to funding.

#### Research capacity building: Training and skill development

Training of researchers in LMICs is critical for the success and sustainability of international health research in Africa [40]. Our interviewees confirmed that this was one of the key features of the genomics research and biobanking initiatives they were involved in. Also, researchers who were part of H3Africa or had knowledge of H3Africa activities, identified the training of the next generation of African scientists as one of the main benefits of the collaboration.

In terms of building capacity, training and giving opportunities for many African students. So that will accelerate capacity within Africa. (R-05)

Training of researchers within these consortia are taking place in institutions in both LMICs and HICs One of our interviewees mentioned that in their project some of the students are being trained out of their home countries and this gives them the opportunity to expand their skills and research networks.

We currently have two trainees from Country Y [LMIC] at Country Z [HIC] at the Z college of Medicine, doing their rotation of laboratories towards their PhDs in genomic sciences and genomic medicine. We also have three from Country X [LMIC] and we are expecting two more from Country Y [LMIC] next year. (R-02)

Another form of human capacity building that has been part of the activities of H3Africa and which more than three quarters of the interviewees identified as a major benefit, is postdoctoral skills development, including a focus on transferable skills. These included training in laboratory techniques, statistics, data analysis and grant writing.

Capacity building in our research to help young African researchers, develop themselves in many fields related to research: statistic, epidemiology……Through this project, I have developed some skills in writing grant proposals (R-09)

Genomics research and biobanking are relatively new in Africa. Many researchers who now work in this field did not receive formal training in genetics, genomics or associated fields such as bioinformatics. In fact, many of our interviewees, and of the H3Africa researchers more generally, are medical doctors with a strong research record in investigating particular diseases. Whilst being world-experts on these diseases, they do not necessarily have expertise in genomics. Training in key genomic research skills such as for instance those relating to bioinformatics is essential for establishing successful and equitable partnerships in genomics research and biobanking agenda in Africa [42]. The sustainability of such training is a key concern, however. One researcher described that his involvement in H3Africa has provided an opportunity for their research team, together with their collaborators in HICs, to develop degree programs in genomics at African universities as a way of ensuring sustainability.

We are working with the Faculty of Health Sciences in both Country X [LMIC] and Country Y [LMIC] to create masters programs, PhD programs in genomics and bioinformatics. We hope that what will be a lasting and sustainable benefit to those countries, is by having a local program in genetics and genomics (R-02)

Although just one of our interviewees mentioned this as a sustainable capacity building effort within H3Africa, it is one that will be worth emulating and should be extended to other African countries. Research capacity building strategies aimed at developing undergraduate and postgraduate research programs in African universities and research institutions are vital in sustaining health research in Africa [40, 48].

The third and last form of training and skills development articulated by almost a third of our interviewees was capacity building in research ethics at both the professional and institutional level. This benefit was only mentioned by H3Africa researchers and they were of the opinion that there has been some improvement in research oversight for genomics research and biobanking in Africa as a result of H3Africa activities.

At the professional level, it has made African biomedical researchers to think beyond the science of their projects to the ethical issues raised by their research projects. Typically, African researchers have demonstrated a knowledge gap in research ethics [49] and so engaging them in research ethics debates may help improve research oversight in Africa

I can almost be sure that if you visited any study site at the beginning of H3Africa and visit them now, the way the will be consenting and engaging with the community will be completely different, I mean in ethics, they have been challenged to think far beyond just the compensation for travel, for time and everything … even our ethics committees have been touched to think far more (R-13)

Weak research oversight systems in Africa challenge the execution of health research projects in Africa [50–52] and make African researchers and research participants more vulnerable to exploitation. Genomics research and biobanking in particular raise unique ethical challenges [53–59] that may be quite different from other forms of international health research such as clinical trials that ethics committees review more habitually. Furthermore, methods of genomics research and biobanking are relatively new to REC members in Africa [55, 57, 60] and they may not be fully equipped to review such studies. It is therefore important that for international health research projects that will involve sharing of samples and data and that raise complex ethical issues, ethics committee members be appropriately engaged in discussions on genomics research and biobanking. One interviewee mentioned that as a result of meetings organised by H3Africa, research ethics committee members in Africa are becoming familiar with concepts in genomics research and biobanking. In his/her opinion, this had implications in research oversight for genomics research and biobanking in Africa.

Another thing which is already happening that is good is involving the ethical review committees in giving them additional training in genomics research because that is a new area for a lot of the IRBs. In Country X we found that the IRBs had not reviewed this type of research before and this caused a lot of delay in terms of getting approvals and feedback from them but things improved after one of the IRB members attended the last consortium meeting for the special session for ethical review committees. (R-02)

Capacity building in research ethics has impact on regulation of health research in Africa as well as the protection of research participants. Africa has historically been characterised by a history of “parachute” or “postal” research whereby researchers from HICs have come to Africa just for the samples and then “disappear” once samples have been collected [38, 61]. This has led to research ethics committees being overly cautious when reviewing projects and in some cases it has led to tight national regulation for the export of samples. It will appear that genomics and biobanking initiatives in Africa are trying to engage research ethics committees in Africa and to build their capacity to be able to identify ELSIs pertinent to genomics research and biobanking. Infrastructural research capacity building.

A second major form of capacity building articulated by all our interviewees is infrastructural capacity building, mainly the setting up of biobanks and the acquisition of laboratory equipment. For these researchers, having a biobank in Africa will be a great resource for biomedical research in Africa.

It [consortium name] has really emphasized on building infrastructure in Africa, giving Africans the tools to solve their problems.....One big advantage of having a biobank in Africa is that it really puts all of our resources together. I think that is very huge for Africa, to begin with. And it is a way of putting our resources together and giving us the infrastructure to produce high quality research (R-13)

Biobanks are repositories where organized collections of human biological materials, and associated data from large numbers of individuals, are collected, stored and distributed for the purpose of scientific investigations or public health use [8, 62]. Biobanks are therefore an important resource for biomedical research. Also, well curated biobanks in Africa could foster international health research collaborations, south-south collaborations and promote biomedical research in Africa and globally [5, 9]. However, for researchers in Africa to make optimal use of the samples and data stored in the biobanks, they will have to secure the necessary laboratory equipment. One of our interviewees mentioned that their genomics research and biobanking project has given them an opportunity to source for recent and novel laboratory equipment.

In terms of infrastructure, we are building capacity at Q University and university of Country X [LMIC] to do genome sequencing and to get their sequencers, their Illumina sequencer and so all of those tools will help in the building of infrastructure in the different laboratories. (R-02)

Besides the modest infrastructural capacity for genomics research and Biobanking in Africa, limited access to technology remains a serious impediment to biomedical research in Africa [63–65]. It is also a major reason for the export of samples from Africa to HIC, especially when high-throughput technology is required [66, 67]. To minimize export of samples, researchers in Africa will need to acquire up-to date laboratory equipment. As one interviewee explained, African countries must emulate LMICs such as India and China and build their infrastructural capacity for genomics research and biobanking.

We should not just be thinking that anything we want to do, we have to collect samples and send to Europe, samples to China, samples to America. We need to build the capacity that we can do work in Africa. If that is the case then we can produce things that will be valuable and useable in Africa. And I think that is just what has been done in several other countries like India. It is very difficult now to take anything out of India for analysis. (R-14)

Export of samples from Africa is a major contributor to exploitation of African researchers. The acquisition of laboratory equipment that will permit research work to be done in Africa will minimize this risk–but only if equipment is maintained. Unfortunately, in many African research institutions, laboratory equipment which are primarily obtained through funds from international health research tend to lie loose with little use once the projects they were obtained for are over. In the unfortunate case of a breakdown, equipment worth thousands of dollars are usually abandoned to the dust for sheer of lack of resources to maintain them. The virtual absence of an infrastructural maintenance culture by research institutions in Africa (both in providing resources and tools required for maintenance) and the impact it has on health research cannot be missed even by a passing observer.

#### Access to funding

The last and major form of research capacity building is sustainable access to funding for health research. This was mentioned by almost half of the interviewees. For these interviewees, being part of a funded genomics research and biobanking consortium has been a unique opportunity to access and administer funds for large-scale research projects.

For researchers, what it is really doing is, it’s building their capacity to do research, to do large scale research that they haven’t done before because it costs a lot of money to do this type of research. (R-15)

Research funding is a major driver of change for biomedical research [68]. A dearth of funding for health research coupled with the lack of research infrastructure (laboratories, biorepositories, databases has held back African scientists from carrying out rigorous health research [48, 69]. The importance of funding health research in Africa has been presented to African governments [70] and while there was a general buy-in by African governments followed by a commitment to allocate a small proportion of their annual national growth domestic product (GDP) to research, there has been little compliance by a vast majority of African governments. Where there has been government commitment, such as in South Africa, tremendous progress has been made in advancing health research. African governments will have to take responsibility and devise research funding schemes that can sustain and foster health research in Africa. One African researcher and a pioneer in research capacity building in Africa has suggested that a Pan-African research funding agency may solve the problem of limited local funding for health research [69]. Organizations like the Alliance for Accelerating Excellence in Science in Africa (http://aesa.ac.ke/), a new partnership between the African Academy of Sciences and the New Partnership for Africa’s Development (NEPAD) may play the role of such a Pan-African funding agency especially as it is acting as an agenda setting and funding platform to address Africa’s health and development challenges. However, it is still funded by the Wellcome Trust and may therefore face the crisis of non-sustainability should there be no financial commitment from African governments and the private sector in Africa to support its activities.

### Challenges in building capacity for genomics research and biobanking in Africa

While research capacity building could be a major way of achieving fairness in international health research partnerships in Africa, it is hard to sustain research capacity building efforts and to retain human research capital in Africa [40]. Brain drain, for example, is a major impediment to research capacity building efforts in Africa [68, 71]. About a third of our interviewees expressed concerns that although young African scientists are being trained in genomics research and biobanking, the lack of an enabling research environment in Africa could see them migrating to research institutions in HICs. Some interviewees suggested that assurances of a research career in African institutions could curb brain drain. One interviewee explained how their project is working towards overcoming the problem of brain drain

We want these trainees to come back and do research that benefits their own countries. And so there is an agreement with the host institutions that there will be faculty positions available for them when they complete their degrees, so they will be able to go straight to those faculty positions and to be able to use the infrastructure that we have been building in the meantime to continue their genomic research, to apply for additional grants and to nurture their own students one day. (R-02)

Generating a pool of qualified African researchers must be accompanied by a parallel interest in maintaining them in research institutions in Africa. Considering that most research institutions in Africa are government establishments, African governments will have to play a key role in retaining emerging researchers in Africa. And whilst African governments may find it hard to direct funds for research activities, they can at least provide research jobs and research support for emerging African researchers.

Equally, whilst the establishment of well-curated biobanks in Africa is one of the greatest benefits of these genomics research and biobanking consortia, maintaining them will be a serious challenge for host institutions. To solve the problem of sustainability a few interviewees again suggested that African governments and the private sector in Africa invest in health research

What I know will be essential will be for African governments to invest more in biomedical research and development at all levels and for African governments and the private sector to improve the health research systems that are currently in place in many African countries. Those efforts will benefit not just genomic research but all aspects of health research. (R-01)

The quote by the researcher above further highlights the importance of African governments to support health research in Africa as this will enable African researchers to work on local health needs rather than having to rely heavily on external support which could come with the specific research interest and priorities of the funders or collaborators in HICs.

## Discussion and conclusion

International health research has great potential in fostering health research in Africa. It is also a platform for both LMIC and HIC researchers to share expertise and resources for the purpose of advancing scientific discovery and to access funding for health research. However, power imbalances between collaborators in Africa and HICs may hinder successful research partnerships and has been known to lead to the exploitation of African researchers and research participants. In this study, we document the results of a qualitative study that aimed at exploring African researchers’ perceptions and expectations of the risks and benefits of international health research collaborations, with a particular focus on genomics research and biobanking in Africa. All the interviewees acknowledged that the opportunity to collaborate and access funding is a benefit for African researchers. However, they expressed fears that may be exploited within these collaborations. In their opinion, fears of exploitation may be minimized through setting up equitable research collaborations and building capacity for genomics research and biobanking in Africa. In this paper, we document that despite persisting fears of exploitation of African scientists, all interviewees were of the opinion that the international genomics research and Biobanking consortia in Africa have provided African researchers with a platform to build their capacity to conduct cutting edge genomics research.

There is a growing acceptance of the importance of research capacity building and African leadership and ownership of health research in Africa as a means of achieving equitable international health research partnerships. The assumption is that equitable research partnerships would ultimately stand a better chance of building trust between research partners and in fostering health research on African health problems, thereby reducing global health inequities. Extrapolating this trend to genomics research and biobanking in Africa, it is equally important to ensure that international collaborations in African genomics research are fair and equitable–and the rhetoric surrounding these initiatives reflects this trend. Some genomics research and biobanking initiatives in Africa such as the H3Africa Consortium have gone some way in defining some of the aspects of fair and equitable international collaboration in African genomics research [9] and to speculate that H3Africa may be setting a gold standard for how collaborative international health research in Africa should be done to benefit African populations [72]. In this study, we go beyond this documented promise to explore African researchers’ perceptions and expectations of benefits and risks of Africa’s participation in collaborative genomics research and biobanking in Africa. For our interviewees, a key hallmark of equitable collaboration is equip African researchers to make equivalent intellectual contributions to the design and conduct of genomics research through a rigorous process of research capacity building. This may take several forms including training of Masters and PhD students, skills development for postdoctoral scientists, mentorship, peer support through continental networks and infrastructural support.

Different forms of research capacity building have been described in the literature [40, 68, 71] and one key component that emerged from the interviews, that goes beyond individual training, is the creation of postgraduate degree programs in genomics in African universities. This approach could foster sustainability of genomics initiatives in Africa [40, 42, 48]. Such degree programs could be designed to meet current needs of African genomics research and need to include training in bioinformatics, genomics medicine, genetic counselling, Bioethics and the social sciences. Equally important in the sustainability of capacity building efforts is the creation of networks that could foster interdisciplinary research in Africa. The H3Africa bioinformatics network (H3ABionet) is an example of a network that provides peer support for bioinformatics research in Africa with nodes in more than 30 different African countries [24]. Such an approach will have to be adopted especially in emerging disciplinary areas that provide support for genomics research and for which there is limited capacity. Examples include genetic counselling, bioethics, sociology and anthropology. Also, because research is a complex activity and process that requires the interplay of individuals, organizations, national and international research systems. Genomics research and biobanking initiatives in Africa will also need to expand their capacity building efforts to include the establishment of centers of excellence for genomics research in Africa, supporting research administration in Africa, strengthening capacity for research-to-policy and promoting public education in genomics in Africa. This will be particularly crucial if these initiatives are to achieve their aim of being an exemplary model for collaborative health research in Africa.

A challenge to the H3Africa experience is that it is based on a set of shared, yet often unarticulated, values and principles that seek to promote equity and fairness. Whilst these principles are incorporated into the design of projects and policies of the H3Africa Consortium, they are not necessarily visible to or shared by other initiatives that support genomics research in Africa. A question is how the H3Africa infrastructure could be effective in influencing the design of such initiatives, to ensure that the principles of fairness and equity are also woven into such future endeavors. It is possible that this could be achieved through for instance the African Academy for Sciences (AAS) and its affiliate organization the Alliance for the Acceleration of Excellence in Science in Africa, but only if there is active advocacy on behalf of the African genomics community, including H3Africa researchers, for this to be the case.

## Supporting information

S1 File Interview guide for researchers. (DOCX)
