# Supplementary material for: Equity in international health research collaborations in Africa: Perceptions and expectations of African researchers
Source: PLoS One. 2017 Oct 16;12(10):e0186237. doi: 10.1371/journal.pone.0186237 (PMC5643046; doi:10.1371/journal.pone.0186237)
Supplement: S1 File — (DOCX) [file pone.0186237.s001.docx]

**Topic Guide for Interviews with Researchers**

1. **Background**
2. So just quickly, could you briefly describe me your H3Africa research project
3. What is your current position in the project (P.I, Co P.I)
4. How long have you been involved in genetic and genomic research
5. **Benefits of Genomic research**
6. What do you think are some of the benefits of genomic research to Africa? ( clinical, patients, your national population, research participants, health care)
7. What are the specific benefits of the genomic research you are currently involved in?
8. How could these benefits be made more practical? Whose responsibility
9. At what stage of the research project should cited benefits be made available?
10. In explaining/describing benefits to participants and to ethics committees- which of the cited benefits are you more likely to talk about?
11. **Clinical Applications**
12. Can you envisage the use of genomics in the clinic?
13. How do you think this could be feasible in Africa?
14. Whose responsibility
15. Do you have any specific examples of how genomics is being used in a clinical setting within Africa or in your country?
16. **Data /sample Sharing**
17. Does the project involve sample/data sharing? (What type?)
18. What are the benefits of data and sample sharing? How will this benefit Africa (participants, researchers etc)
19. What do you feel about data and sample sharing? Is it important?
20. Examples of research that should not be done on samples/data originating from your research?
21. What do you think are some of the risks of data and sample sharing to Africa
22. **Benefit Sharing**
23. Have you come across the term benefit sharing?
24. What does it mean to you?
25. Do you think it is an important concept in genomic research? Africa? why
26. How do you think this concept could be addressed more in genomic research?

Do you think that there is a chance that genomic research may affect African communities? How?

1. **Closing Section**
2. Considering all that we have discussed, how do you think genomic research could be made more beneficial to Africa?
3. Do you think there is need for further discussions on benefits and benefit sharing in genomic research?
4. Anything else they would like to add?
5. Any questions about further research process, timeline etc?
6. Interested in research results? (permission to re-contact)
